# The differential gut microbiota and their MetaCyc pathways in IBRV infected Angus calves

**DOI:** 10.3389/fmicb.2025.1588341

**Published:** 2025-08-29

**Authors:** Pengfei Yi, Tianqing Li, Lianping Xu, Xin Li, Haiyan Wang, Yingcai Ma, Yunxiao Ma, Yawei Sun, Na Li, Qi Zhong, Xuelian Ma, Gang Yao

**Affiliations:** ^1^College of Veterinary Medicine, Xinjiang Agricultural University, Ürümqi, China; ^2^Xinjiang Key Laboratory of New Drug Research and Development for Herbivores, Ürümqi, China; ^3^Animal Disease Control and Diagnosis Center of Bayingolin Mongol Autonomous Prefecture, Korla, China; ^4^Animal Disease Control and Diagnosis Center of Altay Prefecture, Altay, China; ^5^Institute of Veterinary Research, Xinjiang Academy of Animal Science, Ürümqi, China

**Keywords:** infectious bovine rhinotracheitis virus, Angus calves, 16S rRNA, gut microbiota, metabolic pathways

## Abstract

Infectious bovine rhinotracheitis virus (IBRV) is a globally prevalent pathogen that causes respiratory disease in cattle. Emerging evidence suggests that specific bacterial taxa and gut microbial community compositions are strongly associated with viral pathogenesis, by either enhancing or mitigating disease outcomes. This not only impacts the host’s gastrointestinal physiology but also affects distant organs, including the lungs, liver, and brain. However, the impact of IBRV infection on changes in gut microbiota composition and its association with MetaCyc metabolic pathways remains poorly understood. In this study, based on an epidemiological survey of one-month-old Angus calves in a large-scale Angus beef cattle breeding farm consists of four breeding areas located in Maigaiti County of Kashi Prefecture, China. Alterations in the gut microbiota of 10 IBRV-infected Angus calves (IBRV-positive group, P) compared with their 10 healthy counterparts (IBRV-negative group, N), as well as their correlations with MetaCyc metabolic pathways, were investigated using 16S rRNA sequencing. In comparison with N, both Simpson, Shannon and Pielou_e indices of alpha diversity were elevated in P, and the beta diversity showed a marked separation between N and P. The relative abundance of phylum Firmicutes_C was significantly increased, whereas that of phyla Bacteroidota, Cyanobacteria, and Firmicutes_D were reduced in P. The relative abundance of Genera *Dialister* and *Klebsiella* were enriched, while that of *Lactobacillus* and *Blautia_A* were depleted in P. Four distinct MetaCyc metabolic pathways were significantly altered, DENITRIFICATION-PWY, PWY-6906, and P101-PWY were significantly decreased in P, while PWY-7446 was significantly increased. Correlation analysis showed that in N, *Faecalimonas* was positively correlated with both P101-PWY and PWY-6906, and *Limousia* was positively correlated with P101-PWY. *Faecalimonas* was positively correlated with PWY-7446, and *Klebsiella* was positively correlated with DENITRIFICATION-PWY in P. Our results reveal that IBRV infection is associated with significant changes in the gut microbial community and its predicted metabolic functions, which may be linked to the host’s systemic response to the infection. This study provides preliminary data on the association between IBRV infection and gut microbiota profiles, laying a theoretical foundation for future investigations into IBRV pathogenesis and potential targeted prevention strategies.

## 1 Introduction

Infectious Bovine Rhinotracheitis Virus (IBRV), also known as Bovine Herpesvirus Type 1 (BHV-1), is a highly contagious virus that causes severe respiratory infections in cattle (Pardon et al., 2011; Wedgwood et al., 2020). Taxonomically, BHV-1 belongs to the subfamily Alphaherpesvirinae and the genus *Varicellovirus* (Nandi et al., 2009; Biswas et al., 2013). The respiratory symptoms is the most prevalent clinical manifestation of IBRV infection, primarily affecting the nasal cavity and trachea. Early-stage symptoms typically include fever, lethargy, anorexia, dyspnea, excessive nasal discharge, nasal mucosal congestion and ulceration, and swelling of the nasal planum (Nandi et al., 2009; Guo JunQing et al., 2019). In the absence of secondary infections, the disease is usually self-limiting and responds well to treatment. However, recovered cattle remain latent carriers of the virus and can continue to shed it into the environment (Schudel et al., 1986). In cases where symptoms are severe, recovery is challenging, and mortality rates are high (Epj, 1977). Additionally, immunosuppression is a significant characteristic of IBRV infection, which increases the risk of secondary infections and results in substantial indirect economic losses (Wang et al., 2024). Currently, there are no specific antiviral drugs for the treatment of this disease; vaccination remains the primary strategy for prevention and control (Ma et al., 2024).

The gut microbiota comprises a diverse and abundant community of microorganisms residing in the gastrointestinal tract (de Abreu Nascimento et al., 2023). The dynamic interactions between the gut microbiota and the host form a complex ecosystem that plays a vital role in maintaining host health (Barathan et al., 2024). In healthy individuals, the gut microbiota maintains homeostasis through a delicate balance with the host and its external environment (Obata and Pachnis, 2016). However, disruptions caused by factors such as weaning, dietary changes, and environmental alterations can lead to gut microbiota dysbiosis, which has been associated with a variety of diseases, including gastrointestinal disorders, metabolic syndromes, and cancers (Caserta et al., 2023; Tian et al., 2024). Research indicates that gut microbiota is involved in the pathogenesis of various pathogens, with dysbiosis potentially impairing immune responses and increasing the risk of pathogen infections (Pickard et al., 2017). Moreover, gut microbiota is also associated with the pathogenesis of non-gastrointestinal viruses. Mohammed et al. observed an association between gut microbiota dysbiosis and Hepatitis C virus infection, suggesting that microbial alterations may be involved in the disease process (El-Mowafy et al., 2021). Xing et al. (2022) demonstrated that gut microbiota plays a significant role in modulating immune responses to influenza virus infections. Recent reports suggest a close relationship between gut microbiota and the pathogenesis of human immunodeficiency virus and respiratory syncytial virus (Geng et al., 2020; Ji et al., 2021). Also, changes in the composition of certain microorganisms or changes in microbiota-host interactions may increase the susceptibility of mice to bovine viral diarrhea virus (Zhang et al., 2024). Infectious Bovine Rhinotracheitis Virus (IBRV) is a common respiratory virus in cattle, however the correlation between gut microbiota changes and IBRV infection has not been reported. Furthermore, studies have shown that various viruses, such as influenza virus, measles virus, and hepatitis virus, can significantly disrupt host cell metabolic responses by altering carbon, lipid, and amino acid metabolic pathways during infection (Cheng et al., 2020). The MetaCyc database is a resource providing extensive information on metabolic pathways from various organisms that may offer greater detail and specificity for certain metabolic pathways or networks than KEGG pathways do. (Karp et al., 2009; Caspi et al., 2017). Therefore, this study aims to investigate the alterations in gut microbiota in calves infected with IBRV and their association with MetaCyc metabolic pathways, in order to provide a foundation and theoretical basis for future research on the pathogenesis of respiratory viral diseases in calves.

## 2 Materials and methods

### 2.1 Epidemiological survey of respiratory diseases in calves

In 2021, a one-year epidemiological survey of respiratory diseases focusing on one-month-old calves was conducted in a large Angus beef cattle breeding farm consists of four breeding areas located in Maigaiti County of Kashi Prefecture, China. The calf clinical symptoms related to respiratory diseases included fever (rectal temperature above 39.5 °C), coughing (dry or wet cough, exacerbated by activity), and dyspnea (extended head and neck, labored breathing, pronounced abdominal breathing, and accompanying swallowing movements). The study protocol was approved by the Animal Ethics Committee of Xinjiang Agricultural University (2020036 and 2024023).

#### 2.1.1 Sampling

Nasal and fecal samples were collected using sterile swabs inserted deeply into the nasal cavities and rectums from one-month-old calves showing clinical symptoms related to respiratory diseases, as well as randomly from healthy counterparts without the aforementioned respiratory symptoms. The samples were then sealed in cryovials and preserved in liquid nitrogen for future use.

#### 2.1.2 PCR detection for IBRV

The UL27 gene sequence of IBRV deposited in GenBank (Accession No.: MK654723.1) was analyzed using DNAstar, DNAman, and online tools such as IEDB and Bepipred. Amino acid residues from positions 273 to 393, identified with high antigenicity, were selected as the target region. Primers were designed using Primer 5.0 and synthesized by Shanghai Bioengineering Co., Ltd. The primer sequences were F: 5′-CTCTACCGCACGGGCACCT-3′ and R: 5′-GCGGCTCTCGTCTCGCA-3′. The target gene (360 bp) was amplified using a total reaction volume of 15 μL. The reaction conditions were 95 °C for 5 min; 35 cycles of 95 °C for 30 s, 58 °C for 30 s, and 72 °C for 30 s followed by a final extension at 72 °C for 10 min.

### 2.2 Gut microbiota analysis by 16S rRNA gene sequencing

#### 2.2.1 Grouping

Based on the results of PCR testing, 10 fecal samples from calves exhibiting respiratory clinical symptoms and then identified as positive solely for IBRV were randomly selected as the IBRV-positive group (P). Similarly, 10 fecal samples from healthy calves, identified as negative for IBRV and other respiratory viruses, were assigned to the IBRV-negative group (N).

#### 2.2.2 16S rRNA gene sequencing

Genomic DNA was extracted using the Omega Viral DNA Quick Extraction Kit (Rehner et al., 2022). DNA concentration and purity were measured using a spectrophotometer, and the quality of the extracted DNA was assessed by agarose gel electrophoresis. The target fragments were then amplified by PCR. The amplified products were analyzed using 1.2% agarose gel electrophoresis, and the target fragments were recovered using the Axygen Gel Recovery Kit. Based on preliminary quantification results from electrophoresis, the recovered PCR products were subjected to quantitative fluorescence analysis. The V3 and V4 variable regions were selected for paired-end sequencing of community DNA fragments using the Illumina platform. Sequencing services were provided by Shanghai Paisenno Biotechnology Co., Ltd., China.

The Divisive Amplicon Denoising Algorithm 2 (DADA2) method was used for primer removal, quality filtering, denoising, merging, and chimera removal steps (Callahan et al., 2016). Each library was analyzed separately for these steps. R language scripts were used to statistically analyze the length distribution of high-quality sequences across all samples. Each deduplicated sequence generated by DADA2 was referred to as an Amplicon Sequence Variant (ASV). ASV sequences were compared with the Greengenes database (DeSantis et al., 2006), and each ASV in the ASV table was annotated taxonomically at the phylum, class, order, family, and genus levels. After distinguishing the samples, ASV clustering and taxonomic analysis were performed using the Usearch software platform. Alpha-diversity and beta-diversity analyses were conducted using the mothur software based on ASV data, and community structure was statistically analyzed at various taxonomic levels based on taxonomic information. Anosim (Analysis of Similarities) analysis was performed using the vegan package in R programming language.

### 2.3 Data statistics and analysis

Data were presented as mean ± standard error (SE). Differences in the relative abundance of gut microbiota at the phylum and genus levels between the N and P groups were analyzed using the Mann–Whitney test in GraphPad Prism version 10. * Denotes *p* < 0.05, meaning significant difference, ** denotes *p* < 0.01, meaning extremely significant difference. Benjamini-Hochberg false discovery rate (FDR) correction was applied to control for multiple hypothesis testing. Functional predictions based on 16S rRNA gene sequences were performed using the PICRUSt2 pipeline and functionally annotated using the MetaCyc database as a reference (DeSantis et al., 2006). Correlation analysis between the differential genera and significantly changed MetaCyc pathways in the N and P groups was conducted using Spearman’s rank correlation. All correlation analyses and visualizations were performed in R (version 4.4.2) using the ggplot2 and corrplot packages.

## 3 Results

### 3.1 Epidemiological survey of respiratory diseases in calves and IBRV detection

A total of 11,215 calves were born from December 2020 to November 2021, of which 922 exhibited clinical symptoms such as fever, cough, and dyspnea, accounting for 8.2% of the total number of births (922/11,215). Of these, 98 calves died, resulting in a mortality rate of 10.6% (98/922).

A total of 197 calves manifesting clinical symptoms including fever, cough, and dyspnea were randomly sampled from the field. Additionally, 30 calves without clinical symptoms were randomly selected. Nasal and rectal swabs were collected, and PCR testing was performed on nasal swabs for IBRV detection. The results showed that the IBRV positive rate was 23.35% (46/197).

### 3.2 The composition and diversity of the gut microbiota in healthy and IBRV-infected Angus calves

#### 3.2.1 The composition and the differences in the relative abundance of gut microbiota

**Figure 1:**
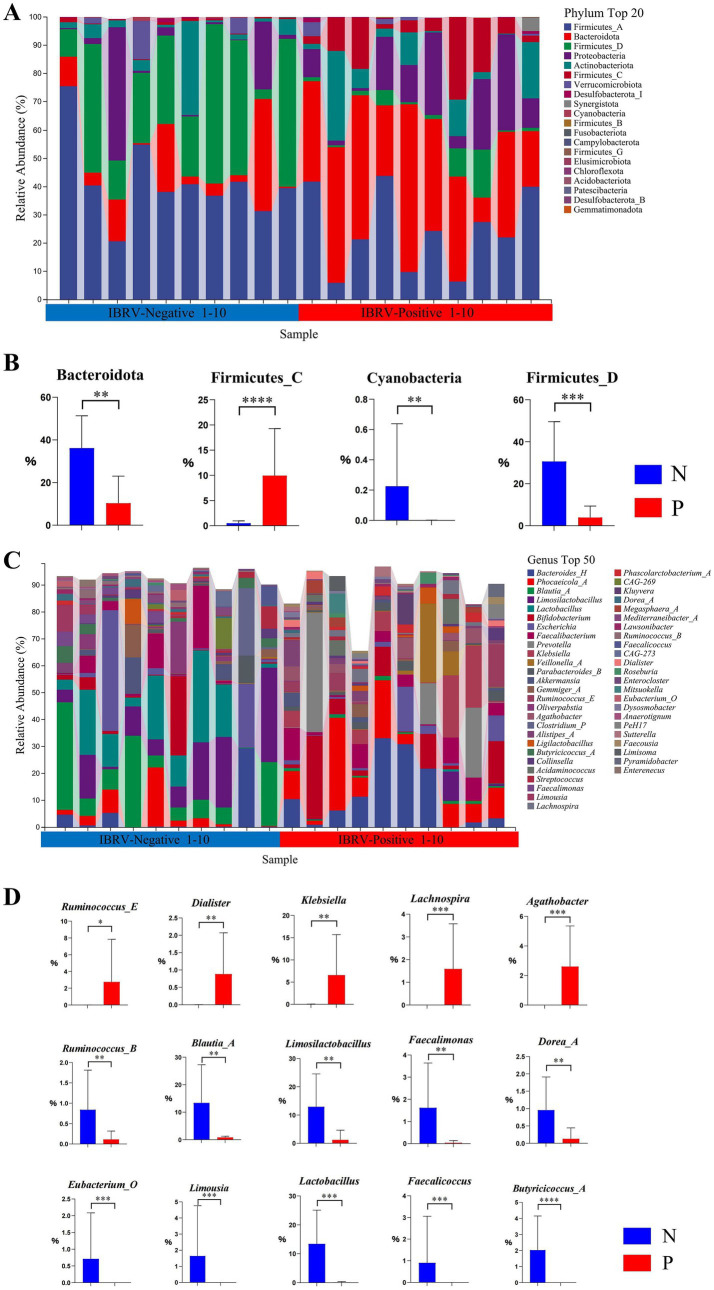
Composition and differential relative abundance of the top 20 phyla **(A,B)** and top 50 genera **(C,D)** between the N and P groups. **(A)** Top 20 phyla based on relative abundance in the N and P groups. **(B)** Differentially abundant phyla between the N and P groups. **(C)** Top 50 genera based on relative abundance in the N and P groups. **(D)** Differentially abundant genera between the N and P groups (N represents IBRV-Negative and P represents IBRV-Positive).

The composition of top 20 phyla and the differentials in the gut microbiota of calves between N and P group were shown in Figures 1A,B, in which there were 4 phyla with significant differences, i.e., in comparison with N group, the relative abundance of Firmicutes_C increased extremely in P group (*p* < 0.01), whereas that of Bacteroidota, Firmicutes_D and Cyanobacteria decreased extremely in P group (*p* < 0.01).

The composition of the genera top 50 and the differentials in the gut microbiota of calves between N and P group were shown in Figures 1C,D, in which there were 15 genera showed significant differences, i.e., in comparison with N group, the relative abundance of *Ruminococcus_E* increased significantly (*p* < 0.05), and that of *Klebsiella*, *Lachnospira*, *Agathobacter* and *Dialister* increased extremely in P group (*p* < 0.01), whereas that of *Ruminococcus_B*, *Blautia_A*, *Limosilactobacillus*, *Faecalimonas*, *Dorea_A*, *Eubacterium_O*, *Limousia*, *Lactobacillus*, *Faecalicoccus*, and *Butyricicoccus_A* decreased extremely in P group (*p* < 0.01).

#### 3.2.2 The diversity of gut microbiota

**Figure 2:**
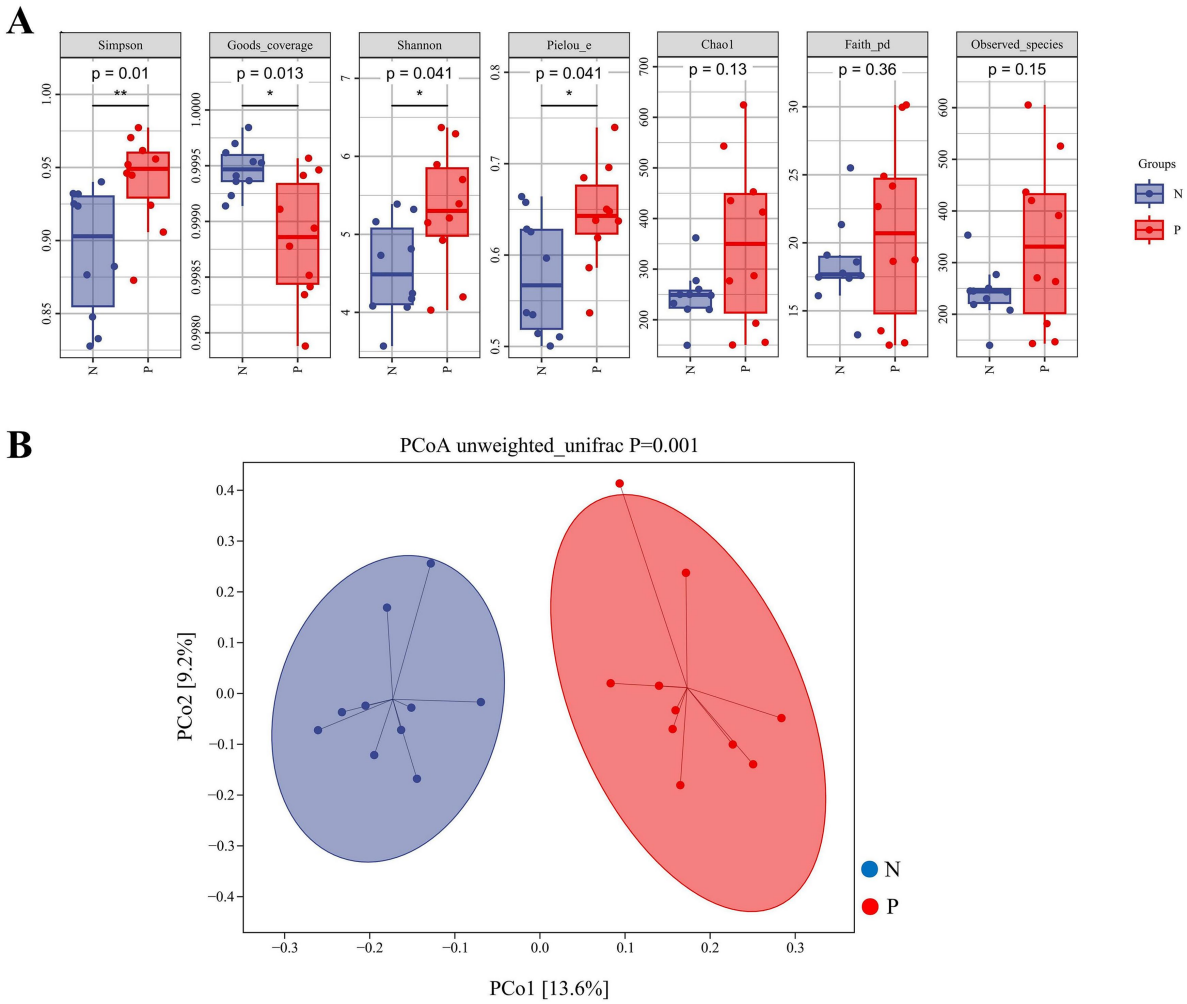
Indices of alpha-diversity **(A)** and beta-diversity **(B)** in the gut microbiota of calves between N and P group. **(A)** Box plots showing alpha diversity indices including Simpson, Shannon, Pielou_e, Chao1, Faith_pd, Observed_species, and Goods_coverage. The vertical axes represent the values of each alpha diversity index. *p*-values were calculated using the Wilcoxon rank-sum test; *p* <  0.05 was considered statistically significant. **(B)** Principal coordinate analysis (PCoA) based on unweighted UniFrac distances. The axes represent the first (PCo1) and second (PCo2) principal coordinates, explaining 13.6 and 9.2% of the variation among samples, respectively. Each dot represents a sample, and the ellipses denote 95% confidence intervals. The group difference was tested by PERMANOVA (*p* = 0.001) (N represents IBRV-Negative and P represents IBRV-Positive).

Alpha and beta diversity of gut microbiota between the N and P groups were shown in Figures 2A,B. The Simpson, Shannon, and Pielou_e indices were significantly higher in the P group than in the N group (*p* < 0.05). In contrast, the Goods_coverage index was significantly lower in the P group than in the N group (*p* = 0.013).

Beta diversity analysis by PCoA revealed that PCo1 was accounted for 13.6% of the variance, and PCo2 accounted for 9.2%. Further Unweighted_unifrac Adonis analysis indicated there was a highly significant independent distribution of gut microbiota between the P group and N group (*p* = 0.001) (Figure 2B).

### 3.3 Functional prediction of gut microbiota

#### 3.3.1 Functional prediction in gut microbiota based on MetaCyc metabolic pathway

**Figure 3:**
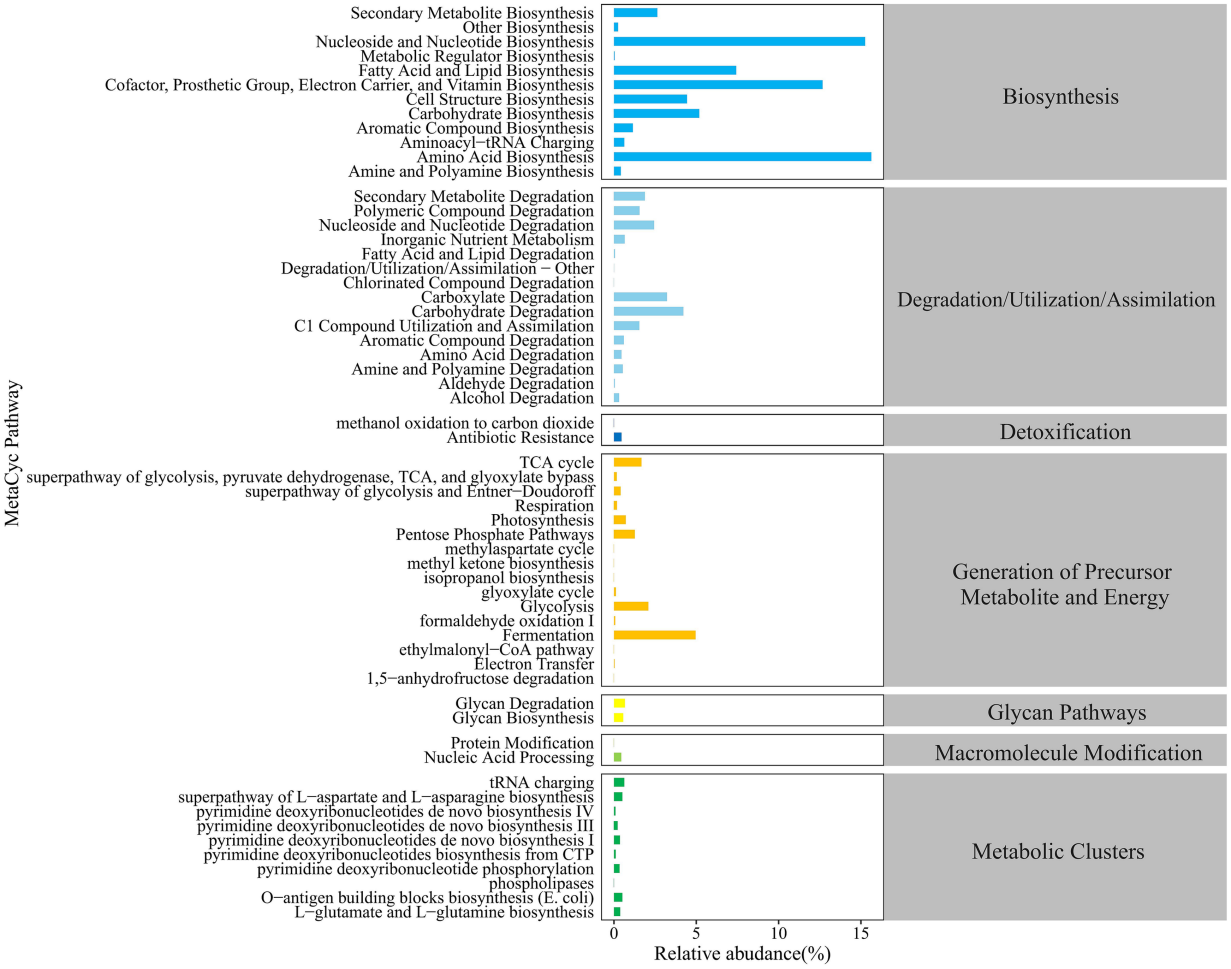
Statistics of MetaCyc metabolic pathways in the Gut Microbiota of calves.

As shown in Figure 3, the MetaCyc database was used to predict the primary metabolic functions of the gut microbiota in groups N and P. The major functional pathways were identified, i.e., biosynthesis, degradation/utilization/assimilation, detoxification, generation of precursor metabolite and energy, glycan pathways, macromolecular modifications, and metabolic clusters. At level 2 in above 7 categories, a total of 59 metabolic pathways were detected, in which pathways with the higher relative abundance were primarily enriched in the biosynthesis, degradation/utilization/assimilation, and the generation of precursor metabolites and energy.

#### 3.3.2 Differential MetaCyc metabolic pathways in the gut microbiota

**Figure 4:**
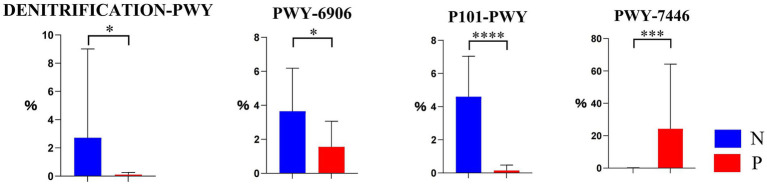
The differential MetaCyc metabolic pathways in the gut microbiota of calves between N and P group. The y-axis indicates the relative functional abundance (%), predicted by PICRUSt2 and normalized by 16S rRNA gene copy number (N represents IBRV-Negative and P represents IBRV-Positive).

As shown in Figure 4, there were 4 differential pathways identified at level 3 in MetaCyc database for function predictions of gut microbiota between the P and N groups. In comparison with group N, the relative abundance of the nitrate reduction I (DENITRIFICATION-PWY) in inorganic nutrient metabolism and respiration, the chitin derivatives degradation (PWY-6906) in carbohydrate degradation, and the ectoine biosynthesis (P101-PWY) in amide, amidine, amine, and polyamine biosynthesis in group P was significantly or extremely decreased (*p* < 0.05 or *p* < 0.01), while that of sulfoquinovose degradation I (PWY-7446) in carbohydrate degradation was extremely increased.

#### 3.3.3 Differential genera in contribution to differential MetaCyc metabolic pathway

A Mann–Whitney test was conducted to compare 15 distinct genera identified in Section 3.2.1 that contribute to the differential MetaCyc metabolic pathways of the gut microbiota in groups N and P. The results revealed significant differences in genera within the ectoine biosynthesis (P101-PWY) and the chitin derivative degradation pathway (PWY-6906) between the two groups. However, no significant genera differences were observed in the nitrate reduction I pathway (DENITRIFICATION-PWY) and the sugar degradation pathway (PWY-7446, sulfoglycolysis).

##### 3.3.3.1 Differential genera in the ectoine biosynthesis pathway

**Figure 5:**
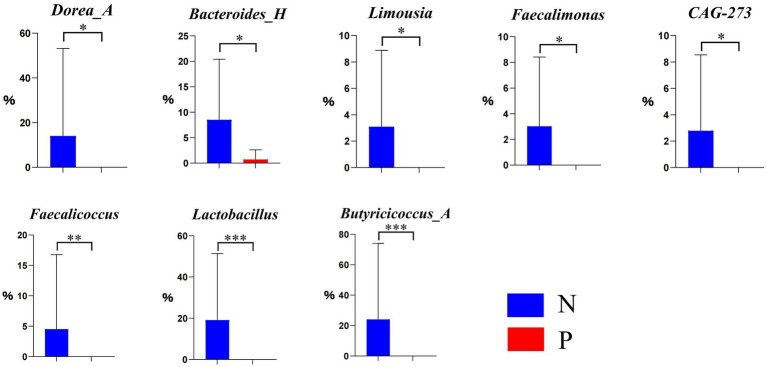
Differential genera in the ectoine biosynthesis pathway P101-PWY between N and P group (N represents IBRV-Negative and P represents IBRV-Positive).

As illustrated in the ectoine biosynthesis (P101-PWY) in Figure 5, the relative abundances of *Dorea_A, Bacteroides_H*, *Limousia*, *Faecalimonas*, and *CAG-273* were significantly lower (*p* < 0.05), and that of *Faecalicoccus*, *Lactobacillus*, and *Butyricicoccus_A* was extremely lower (*p* < 0.01) in group P, if compared to group N.

##### 3.3.3.2 Differential genera in the chitin derivative degradation pathway

**Figure 6:**
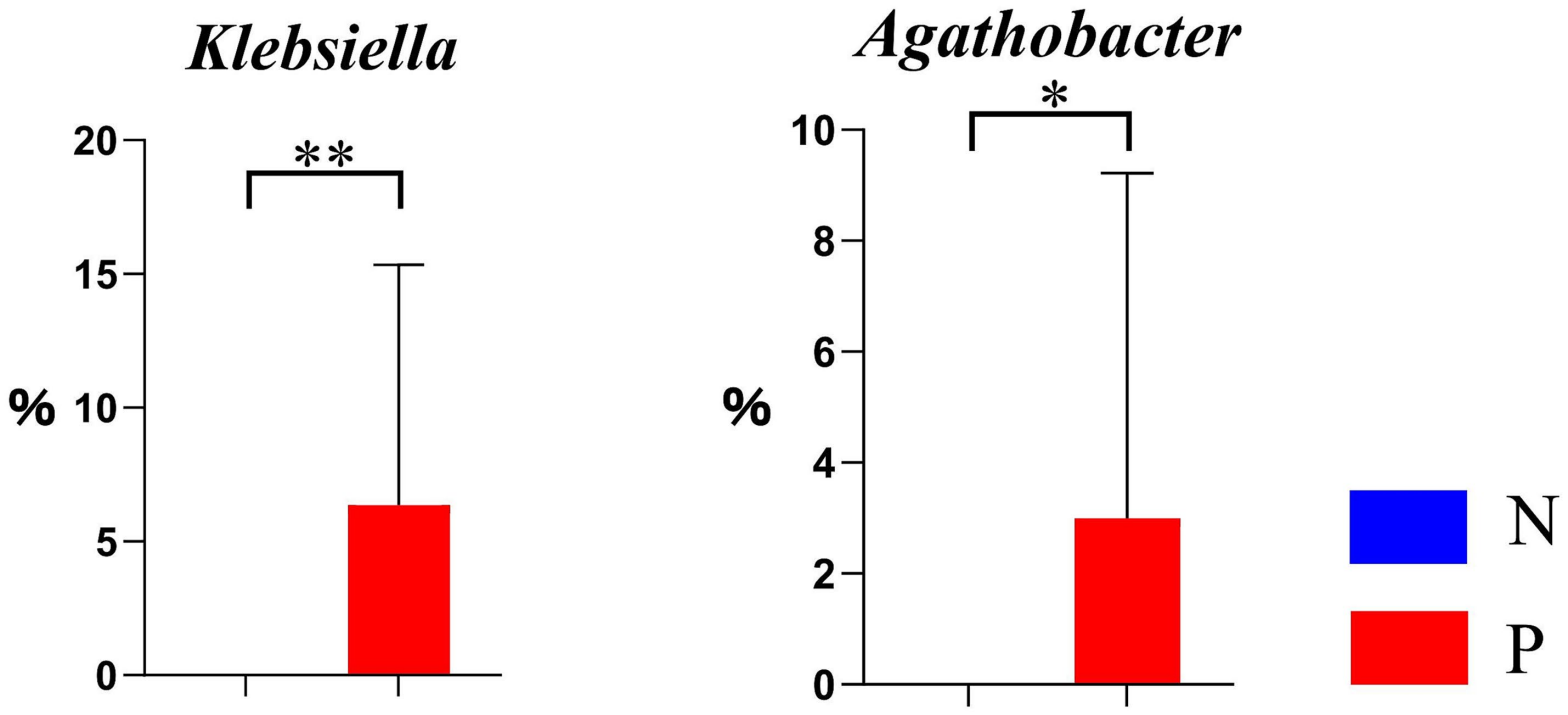
Differential genera in the chitin derivative degradation pathway PWY-6906 between N and P groups (N represents IBRV-Negative and P represents IBRV-Positive).

As illustrated in the chitin derivative degradation pathway (PWY-6906) in Figure 6, the relative abundance of *Agathobacter* was significantly higher (*p* < 0.05), and that of *Klebsiella* was extremely higher (*p* < 0.01) in group P, if compared to group N.

#### 3.3.4 Correlation analysis between differential genera of gut microbiota and differential MetaCyc metabolic pathways

**Figure 7:**
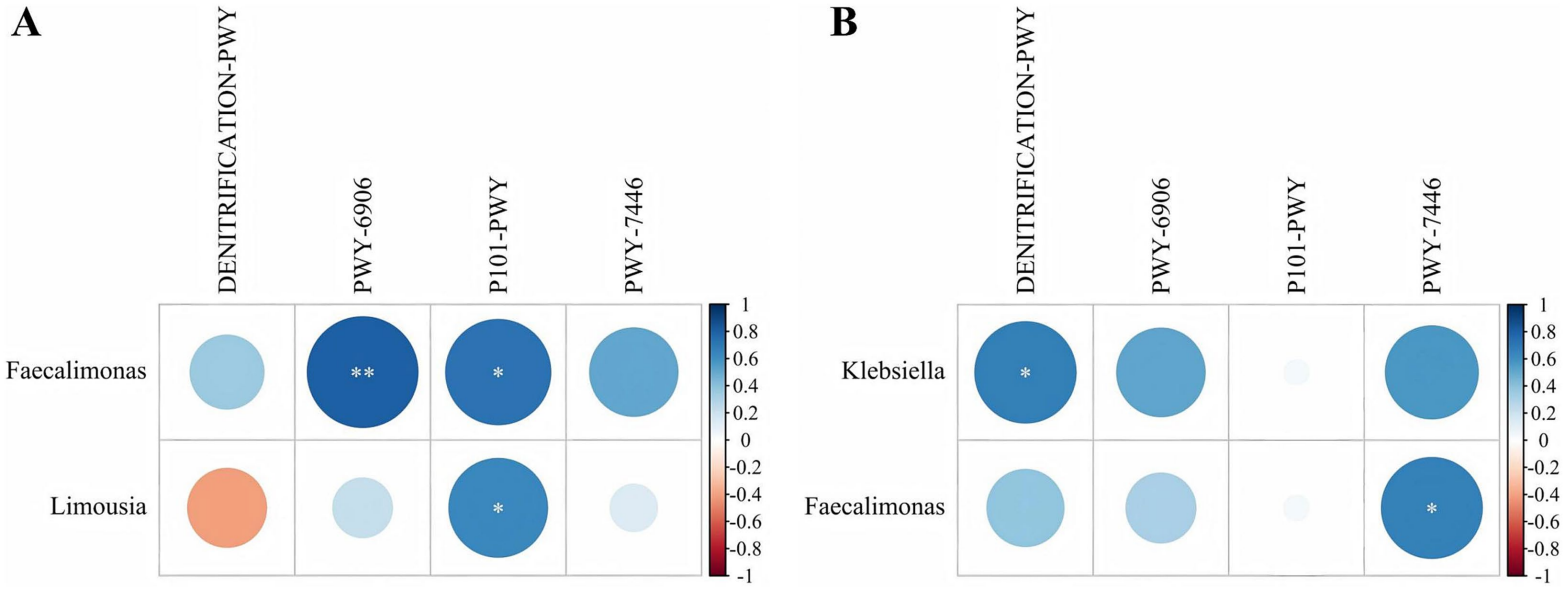
Correlation between the differential genera and differential MetaCyc metabolic pathways in group N and P. **(A)** Group N correlation. **(B)** Group P correlation (N represents IBRV-Negative and P represents IBRV-Positive).

A correlation analysis was conducted between the 15 differentially abundant genera and the 4 differential MetaCyc metabolic pathways. In N group, two differential genera were significantly correlated with two metabolic pathways (Figure 7A). Specifically, *Faecalimonas* was highly significantly positively correlated with the ectoine biosynthesis pathway (P101-PWY) (*p* < 0.05, *r* = 0.736). *Limousia* was significantly positively correlated with the ectoine biosynthesis pathway (*p* < 0.05, *r* = 0.648). Additionally, *Faecalimonas* showed a highly significant positive correlation with the chitin derivatives degradation pathway (PWY-6906) (*p* < 0.01, *r* = 0.815).

In P group, two differential genera were significantly correlated with two metabolic pathways (Figure 7B). *Faecalimonas* showed a significant positively correlation with the same pathway (*p* < 0.05, *r* = 0.679). Additionally, *Klebsiella* was significantly positively correlated with the nitrate reduction I pathway (DENITRIFICATION-PWY) (*p* < 0.05, *r* = 0.683).

## 4 Discussion

Respiratory viral infections are known to influence gut microbiota composition, as reported for influenza and respiratory syncytial virus (RSV) (Haak et al., 2018; Groves et al., 2020). Similarly, SARS-CoV-2 has been shown to disrupt gut microbial ecology and induce intestinal inflammation (Zuo et al., 2020; Li et al., 2021). IBRV infection led to notable changes in the relative abundance of specific phyla and genera, with increases in Bacteroidota, and decreases in Firmicutes_D and Verrucomicrobia. Bacteroidota, an essential component of the gut microbiota, has been linked to inflammation and respiratory diseases; its dysregulation may exacerbate lung inflammation via the gut-lung axis, as seen in asthma patients (Chiu et al., 2021; Zhao et al., 2023). Firmicutes is known to regulate immune function, supporting immune balance under normal conditions, and its reduced abundance may weaken the immune system’s defense against pathogens (Duan et al., 2022). Normally, Verrucomicrobia can modulate host immune responses to a certain extent (Zhang et al., 2022b); its abundance may be altered when viral infections disrupt gut microbiota balance (Zhang et al., 2022a). During rotavirus infections, for example, the gut microbiota ecosystem is disrupted, affecting the growth and metabolism of Verrucomicrobia (Jang et al., 2018). At the genus level, the relative abundance of *Klebsiella* increased, while *Lactobacillus* and *Blautia_A* decreased. *Klebsiella*, which is highly pathogenic, includes *Klebsiella pneumoniae*, the most common species (Conde-Pérez et al., 2024), and some studies suggest that *Klebsiella* may act synergistically with viruses to worsen disease progression (Yasir et al., 2023). *Lactobacillus* enhances immune function by modulating immune cell activity and protects mucosal barriers in the gut and respiratory tract by strengthening tight junction protein expression, thus resisting viral invasion of cells (Peng et al., 2022; Shi et al., 2022). Additionally, *Lactobacillus* can reduce viral infection at colonization sites, such as the gut, through competitive exclusion (Deng et al., 2021; Bu et al., 2023). *Blautia_A* also plays a role in immune regulation, supporting immune homeostasis and enhancing gut barrier function (Mukherjee et al., 2020; Zhao et al., 2022). The results suggest that these changes in the abundance of specific phyla and genera are closely associated with IBRV infection.

Viral infections have been shown to significantly alter the composition and diversity of the gut microbiota. For example, in mouse models of influenza virus infection, the abundance of beneficial gut bacteria decreases while certain opportunistic pathogens increase (Sun et al., 2020). Similarly, changes in gut microbiota diversity and the proportions of specific genera have been observed following SARS-CoV-2 infection (Nguyen et al., 2023). In the present study, alpha diversity analysis revealed that gut microbiota diversity was higher in IBRV-infected calves compared to healthy controls. Beta diversity analysis further demonstrated significant compositional differences between the two groups. Previous studies indicate a dynamic and complex interaction between viral infections and gut microbiota alterations (Groves et al., 2018; Vujkovic-Cvijin and Somsouk, 2019). Viral infections can elicit immune responses that not only target the pathogen but also influence the intestinal environment, thereby reshaping the gut microbial community (Kloepfer and Kennedy, 2023). Such changes may damage the intestinal mucosa, increasing susceptibility to secondary infections (Preveden et al., 2017). Moreover, IBRV infection significantly affected the functional potential of the gut microbiota. Multiple differential MetaCyc metabolic pathways were identified between IBRV-infected and healthy calves, particularly those related to biosynthesis, degradation/utilization/assimilation, precursor metabolite generation, and energy production. These alterations suggest that IBRV infection may influence host physiological processes such as nutrient absorption, metabolic regulation, and immune function (Li et al., 2022; Singh et al., 2024). Notably, nitrate reduction I (DENITRIFICATION-PWY) and chitin derivatives degradation (PWY-6906) pathways were significantly downregulated in the infected group. Nitrate is involved in nitrification and reduction processes critical for nitrogen metabolism in animals (Rizzatti et al., 2017), while chitin metabolism contributes to nitrogen cycling and elemental balance (Deng et al., 2022). Both are essential for maintaining nitrogen homeostasis and energy metabolism. Additionally, the ectoine biosynthesis pathway (P101-PWY) was markedly downregulated in IBRV-infected calves. Ectoine is a protective molecule that helps microorganisms regulate intracellular osmotic pressure and maintain membrane and protein integrity under osmotic stress (Ojeda et al., 2015; Damacena-Angelis et al., 2017). Conversely, the sulfoglycolysis pathway (PWY-7446) was significantly upregulated following IBRV infection. The metabolic products of this pathway, such as butyrate, are beneficial to the host; for instance, butyrate serves as a key energy source for intestinal epithelial cells (Xiao et al., 2023). For instance, butyrate, produced during carbohydrate degradation, serves as a primary energy source for intestinal epithelial cells (Sharma et al., 2021). Furthermore, correlation analysis between the 15 differentially abundant genera and the four differential MetaCyc pathways revealed that *Faecalimonas* showed a highly significant positively correlation with the chitin derivatives degradation pathway (PWY-6906), potentially reflecting its role in maintaining host metabolism (Tlaskalová-Hogenová et al., 2004). Whereas *Faecalimonas* and *Limousia* in the gut microbiota of healthy calves were significantly positively correlated with ectoine biosynthesis (P101-PWY). These genera, commonly found in the gut microbiota, are reported to play roles in immune regulation by promoting mucus secretion from intestinal epithelial cells, which helps maintain the integrity of the gut mucosal barrier (Viggiano et al., 2015; Rastogi and Singh, 2022). These functional alterations suggest that IBRV infection not only changes the structural composition of gut microbiota but also impairs essential microbial functions that help maintain host health. The downregulation of pathways such as ectoine biosynthesis pathway, nitrate reduction I, and chitin degradation indicates a reduced microbial capacity for osmotic protection, nitrogen balance, and barrier support. This may weaken the host’s mucosal defense and immune homeostasis, thereby increasing susceptibility to IBRV infection and exacerbating its progression. Conversely, the upregulation of the sulfoquinovose degradation I pathway may reflect a microbial adaptation to inflammation or dysbiosis. Collectively, these results support a potential mechanistic link between gut microbiota metabolic function and IBRV pathogenesis. In IBRV-infected calves, the ectoine biosynthesis pathway (P101-PWY) was significantly downregulated, with the relative abundances of *Faecalimonas* and *Limousia* in this pathway markedly lower than those in healthy calves. In the gut microbiota of IBRV-infected calves, Klebsiella was significantly positively correlated with the nitrate reduction I pathway (DENITRIFICATION-PWY), whereas *Faecalimonas* was significantly positively correlated with the sulfoglycolysis pathway (PWY-7446), which was not consistent with the increase in relative abundance of *Klebsiella* and decrease in relative abundance of *Faecalimonas* after IBRV infection. This suggests that there may be a discrepancy between changes in abundance of some genera and changes in abundance of metabolic pathways during alterations in the gut microbiota (Li et al., 2023). The exact reasons for this situation are expected to be explored in future study.

## 5 Conclusion

This study demonstrated significant alterations in the gut microbiota of IBRV-infected calves and their association with MetaCyc metabolic pathways. The gut microbiota may play a crucial role in regulating the host’s response to IBRV infection through gut microbial metabolic activities linked to the onset and progression of the disease. Further investigations are warranted to clarify the causal relationship between gut microbiota alterations and IBRV pathogenesis, as well as the mechanisms by which specific bacterial taxa influence the IBRV infection process. It would facilitate the development of strategies for preventing and treating IBRV infection via gut microbiota intervention.

## Data Availability

The original contributions presented in the study are publicly available. This data can be found here: https://www.ncbi.nlm.nih.gov/genbank/, accession number MK654723.1.
